# A consumer register: an acceptable and cost-effective alternative for accessing patient populations

**DOI:** 10.1186/s12874-016-0238-8

**Published:** 2016-10-10

**Authors:** Jamie Bryant, Rob Sanson-Fisher, Elizabeth Fradgley, Breanne Hobden, Alison Zucca, Frans Henskens, Andrew Searles, Brad Webb, Christopher Oldmeadow

**Affiliations:** 1 Health Behaviour Research Group, Priority Research Centre in Health Behaviour and Hunter Medical Research Institute, HMRI Building, University of Newcastle, Callaghan, New South Wales, 2308 Australia; 2 Distributed Computing Research Group; School of Electrical Engineering & Computer Science; Priority Research Centre for Health Behaviour; University of Newcastle, New South Wales, 2308 Australia; 3 Health Research Economics, Hunter Medical Research Institute, New Lambton Heights, New South Wales, 2305 Australia; 4 Hunter Medical Research Institute, New Lambton Heights, New South Wales, 2305 Australia

**Keywords:** Patient recruitment, Consumer participation, Research register

## Abstract

**Background:**

Population-based registries are increasingly used to recruit patient samples for research, however, they have several limitations including low consent and participation rates, and potential selection bias. To improve access to samples for research, the utility of a new model of recruitment termed the ‘Consumer Register’, that allows for direct patient recruitment from hospitals, was examined. This paper reports: (i) consent rates onto the register; (ii) preferred methods and frequency of contact; and (iii) the feasibility of establishing the register, including: (a) cost per person recruited to the register; (b) the differential cost and consent rates of volunteer versus paid data collectors; and (c) participant completion rates.

**Methods:**

A cross-sectional survey was conducted in five outpatient clinics in Australia. Patients were approached by volunteers or paid data collectors and asked to complete a touch-screen electronic survey. Consenting individuals were asked to indicate their willingness and preferences for enrolment onto a research register. Descriptive statistics were used to examine patient preferences and linear regression used to model the success of volunteer versus paid data collectors. The opportunity and financial costs of establishing the register were calculated.

**Results:**

A total of 1947 patients (80.6 %) consented to complete the survey, of which, 1486 (76.3 %) completed the questionnaire. Of the completers, the majority (69.4 %, or 1032 participants) were willing to be listed on the register and preferred to be contacted by email (50.3 %). Almost 39 % of completers were willing to be contacted three or more times in a 12 month period. The annual opportunity cost of resources consumed by the register was valued at $37,187, giving an opportunity cost per person recruited to the register of $36. After amortising fixed costs, the annual financial outlay was $23,004 or $22 per person recruited to the register. Use of volunteer data collectors contributed to an annual saving of $14,183, however paid data collectors achieved significantly higher consent rates. Successful enrolment onto the register was completed for 42 % of the sample.

**Conclusions:**

A Consumer Register is a promising and feasible alternative to population-based registries, with the majority of participants willing to be contacted multiple times via low-resource methods such as email. There is an effectiveness/cost trade off in the use of paid versus volunteer data collectors.

## Background

### Recruitment of adequate and representative samples is critical to methodologically rigorous research

When recruiting individuals to participate in research, two factors are crucial to ensure methodological rigour: that a sample adequately representative of the target population is recruited; and that a sample of sufficient size is recruited to ensure the study is powered to detect statistically significant differences [1]. A significant and well acknowledged challenge for health and medical researchers is obtaining access to large and representative patient samples with minimal selection bias in a timely and cost effective manner [2]. In order to rapidly enhance research quality while simultaneously reducing the time between the development of research ideas and translating results into benefits for the community [3], a shift in the approach to accessing patient populations is vital. System-wide solutions for overcoming access barriers must be prioritised and their feasibility examined.

### Registries as a method of patient recruitment

A register can be defined as “an organized system that uses observational study methods to collect uniform data (clinical and other) to evaluate specified outcomes for a population defined by a particular disease, condition, or exposure and that serves one or more predetermined scientific, clinical or policy purposes” [4]. Various types of registries including population-based and hospital-based registries exist and have been used for different research purposes. Hospital-based registries have primarily been utilised for facilitating clinical care and quality assurance activities [5]. Population-based registries require health services and professionals to record and report all cases of a particular condition. For example, in Australia population - based registries exist to record diagnoses of cancer and cystic fibrosis. This is a powerful mechanism for collecting, analysing and providing access to a variety of patient groups [6–8] and is being increasingly employed to access patient samples for research purposes [2, 9–11]. However, as the primary aim of population-based registries is to monitor disease incidence and mortality, with the goal of health promotion at a population level [12–14], the utility of many population-based registries for enabling access to patients for research purposes is sub-optimal for a number of reasons.

#### Cost

Maintaining a population-based register that collects and verifies complex medical information involves considerable costs. Patient recruitment via population-based registries therefore usually requires researchers to pay a fee to cover recruitment and administrative costs. These costs can involve employing additional medical coders to “fast-track” cases through the register system and costs associated with complex screening and opt-in processes. As research funding becomes increasingly difficult to obtain, researchers may be unable to afford access to sufficiently large samples, potentially limiting the generalizability and rigor of research findings.

#### Delay in access

There are a number of delay and access problems associated with population-based registries. Firstly, there is often a several month delay between disease diagnosis and entry on to the register, preventing researchers from accessing patients close to diagnosis or at the point of contact with health services. If a complex screening process is required, this can also pose a considerable waiting period until permission from both notifying physicians and patients to provide contact details is gained. Secondly, slow and outdated systems of data collection may be another factor influencing this delay, with a recent study examining Australian clinical registries finding that 64 % of registries surveyed used paper based forms to collect patients’ contact details and clinical data and to enrol patients [15]. Updating data collection systems using electronic software is a potential solution to decreasing delays that prevent research being conducted with recently diagnosed patients.

#### Low consent rates

There is potential for inaccurate representation of the population and bias in study results caused by low patient or physician consent rates [2]. This limitation of population-based registries has been recognized, and efforts to improve potential barriers have been undertaken with limited success. For example randomised controlled trials which have used methods such as priming letters to increase recruitment rates [16] have not been successful in significantly increasing rates of consent. These limitations need to be addressed to ensure methodologically rigorous research.

#### Not patient-focussed

Consumer groups and research funding bodies are increasingly advocating for more active involvement in health decision making and service evaluation, heralding the movement to a more informed and shared patient-practitioner model of health care [17, 18]. This is now extending to research involvement, where consumers are viewed as active participants and their preferences on how they would like to contribute to research are incorporated [19]. Many population-based registries require the consent of the patient’s acting physician before the opportunity to participate is offered to the patient [20]. This method does not enable a patient to exercise their right to consent to research participation but rather endorses the physicians’ role as a ‘gatekeeper’ wherein participation is based primarily on their consent. Beskow et al. conducted a cross-sectional survey of 100 colorectal cancer patients’ preferences for research recruitment through cancer registries. This study reported approximately four in five patients (81 %) believed the decision to participate in research should be theirs and not their physicians [21].

#### ‘Consent for Contact’ methods can overcome limitations of population-based registries

One proposed method of engaging consumers in medical research involves gaining generic consent to be contacted about future research studies which are potentially relevant [22]. This method is referred to as ‘Consent for Contact’ or ‘Permission to Contact’. Several studies have documented the implementation of registries which utilise this recruitment method [23–26]. Studies have demonstrated it to be cost efficient [24, 25], accelerate patient involvement in research [23, 24], have high consent rates [23, 25, 26] and to improve consumer autonomy by reducing the role of the clinician as a ‘gatekeeper’ [23]. With this method demonstrating such promising results, there is an urgent need to continue this research through the consideration of consumer preferences for research participation, such as frequency of participation and methods of contact.

### Aims

To overcome the limitations of population-based disease registries in accessing patients for research purposes, a new consumer register was created by the Hunter Medical Research Institute (HMRI). The primary goal of the register is to facilitate access to patient populations for research and advocacy purposes. This proof of concept study examines the feasibility of establishing the HMRI Consumer Register. The aims of this research are to examine:

i. Patient consent rates for recruitment onto the HMRI Consumer Register;
ii. Consumers’ preferences for frequency and mode of contact for receiving an invitation to participate in research through the register;
iii. The feasibility of establishing the register, including: (a) the cost per person recruited onto the register; (b) the differential cost and success of volunteer versus paid data collectors; and (c) participant completion rates.

## Method

### Design

A cross-sectional survey was administered to individuals accessing outpatient specialist services. Data collection occurred from January to December in 2013, and in June and July 2014. In total, 58 weeks of data collection took place. Ethics approval was provided by the Hunter New England Human Research Ethics Committee (12/08/15/4.04) and the University of Newcastle Human Research Ethics Committee (H-2013-0234). Informed consent to participate in the study was obtained from participants.

### Setting

Two regional public teaching hospitals and one private hospital in New South Wales, Australia, participated. The largest of the public hospitals has over 500 in-patient beds, with an average of 530 outpatients seen on a daily basis. Cardiology, neurology, orthopaedics, obstetrics and gynaecology clinics participated from this hospital. Medical oncology patients were recruited from the remaining two hospitals. The public hospital has 195 beds and an average of 300 outpatients seen on a daily basis. The private hospital has 171 in-patient beds and, at the time of participant recruitment, was the largest private hospital in its region.

### Recruitment and training of volunteer data collectors and research assistants

To allow comparisons of costs and success in patient recruitment, both volunteer and paid data collectors were utilised. Volunteer data collectors were recruited from medical-related community organizations. Both groups completed competency-based training involving one two-hour interactive training session which covered data collection procedures, ethical issues surrounding approaching potential participants, and role plays. All volunteers and research assistants completed a supervised patient recruitment session with a member of the research team and had weekly telephone or face-to-face contact with a member of the research team. In addition, all volunteers and research assistants received a monthly newsletter which provided an update about the numbers of participants recruited and consent rates. Volunteers and research assistants were also provided with feedback on their individual consent and completion rates, benchmarked against other volunteers and research assistants, on a monthly basis. Those with consent rates under 70 % were provided with personalised feedback on how to improve their consent rates and offered a booster training session where necessary.

### Participants

Eligible patients were those attending a participating outpatient clinic, aged 18 years and above, able to speak and read English to a level that enabled completion of an English language survey, and physically and mentally well enough to participate. Patients were directly approached by volunteers or research assistants at the point of care, without requiring the permission of their clinicians, to inform them about the register. Patients were provided with an overview of the research, and assessed for eligibility. Eligible patients were provided with an information statement and invited to complete the survey on a touchscreen tablet computer while waiting for their appointment or receiving intravenous treatment. If requested, assistance with the touchscreen technology was provided. Consent was implied based on survey commencement. Gender and clinic of recruitment were recorded for non-consenters.

### Measurement

Participants completed a survey on a touchscreen tablet using a web-based software to allow immediate data availability and allow features to be incorporated to enhance data completeness [27]. To answer questions, participants touched their selected response option using their finger or an attached stylus. The following was collected for each participant:

#### Willingness to be contacted about future research opportunities

**Fig. 1:**
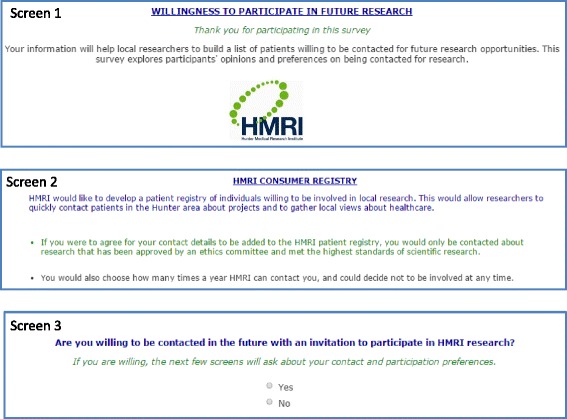
On-screen explanation of the HMRI Consumer Register provided to patients

Willingness to be contacted about future research opportunities was assessed (response options: yes/no). Prior to selecting a response, participants were provided with an on-screen explanation about what agreeing to be contacted would mean (see Fig. 1). Participants who indicated a willingness to be contacted were asked an additional three questions: (i) how many times in the next 12 months they were willing to be contacted (response options: 1, 2, 3, 4, 5, 6 or more); (ii) if they had internet access (response options: yes/no); and, (iii) their preferred methods of such contact (select any of the response options: email, home telephone, mobile telephone and/or post). Only those with internet access were asked if they preferred to receive email invitations.

#### Demographic information

Participants were asked to self-report: age, gender, highest level of education obtained, marital status, Aboriginal and/or Torres Strait Islander origin, private health insurance coverage and if they possessed a concession card.

These questions were embedded within a larger survey assessing patient preferences for health service change. Additionally, volunteers’ and research assistant’s demographic characteristics including age, gender, previous experience in medical settings, were recorded to ascertain any difference in recruitment success.

### Statistical analysis

Differences between those who consented to survey completion and those who did not, and differences between those who consented to be contacted about future research and those who did not, were examined using chi-squared univariate analyses. Descriptive statistics were used to examine patient preferences regarding preferred frequency and acceptable modes of contact. Recruitment rates were calculated for each recruiter as the number successfully recruited out of the number approached, these were compared between recruiter gender, age, medical experience and position (research assistant or volunteer) on the absolute scale using simple linear regression models with bootstrapped standard errors. Separate models were fit for each recruiter characteristic, and a multivariable model including all characteristics was also estimated. Estimated marginal means (and 95 % confidence intervals) are presented for each model, as well as regression coefficients (interpreted as absolute difference in means), 95 % bootstrapped confidence intervals, *p*-values and R-squared values.

### Cost analysis

Costs were assessed by stipulating the resources to be included in the costing; how those resources would be measured; and, how monetary units would be applied [28]. The costing reflects the resources, including overheads, required to approach, recruit and administer the survey to consenting participants. The costing was based on: (i) the economic concept of ‘opportunity cost’, which is the value of activities, or choices, forgone because of the resources committed to the register; and (ii) the ‘financial cost’, which is the monetary outlay on the register. The time of volunteers was included in the opportunity cost model as these hours could have been dedicated to some other function that would benefit the community however, this value was not included in the analysis capturing actual financial outlays. The opportunity cost of an hour of volunteered time was set at the paid interviewer rate which was as a Research Assistant.

Resources considered for the costing were those directly expended, compensated or forgone by HMRI to recruit register participants in 1 year: 2013. These included administration, survey development, information technology as well as training and recruitment. Overheads were applied to paid labour (academic oversight and research assistance) but not to volunteer labour. Overheads include allowances for on-costs of employment such as sick leave, superannuation and annual leave; these do not accrue for volunteers. The time of patients during recruitment surveys was excluded from the costing. Study records supported the use of a bottom-up approach to measure resources committed to the register. A bottom-up approach to costing examines the cost of delivering a process by costing individual segments of activity. Combining these ‘unit’ cost allows an estimate of total cost Monetary values in 2013 Australian dollars were applied to the quantities of resources used to recruit patients to the register. Market prices are an appropriate proxy for opportunity cost [29]. Training was assumed to be a recurrent annual cost and included the labour cost associated with the trainers and, for the opportunity cost estimate, the value of volunteer time. Equipment and touchscreen tablets were included at the purchase price. Hourly wage rates used in the costing were calculated using current professional pay rates at the University of Newcastle. While costs were recorded over 1 year, some fixed costs could be amortised over longer time frames because assets, such as touchscreen tablets, could be utilised over several years. Costs are reported based on total cost in the first year of the register and as an annualised cost which spreads fixed costs over their useful lives.

## Results

### Sample

A total of 2417 patients were approached to complete the tablet survey, of which, 1947 (80.6 %) provided consent for survey completion. Table 1 provides the demographic characteristics of individuals who did and did not provide consent to participate. Of the 470 non-consenters, gender and clinic of recruitment were recorded for 446 (94.9 %). Of the 1,947 participants who consented to complete the tablet survey, gender and clinic was recorded for 1,880 (96.6 %). There were no significant differences in consent according to gender, however consent rate was significantly impacted by clinic of recruitment (*P* =0 .001), with cardiology and neurology patients (83.9 %) and orthopaedics patients (84 %) more likely to agree to participate than oncology or obstetrics and gynaecology patient groups.

**Table 1:** Demographic characteristics of individuals by gender and clinic group according to consent status (*n* = 2,326)

Demographic characteristic	Consent status		Test statistics		
	Consent (*n* = 1880*) n (%)	Non-consent (*n* = 446**) n (%)	*χ* ^*2*^	df	*P*
Gender			*0.23*	*1*	*0.63*
Male	706 (81.3)	162 (18.7)			
Female	1174 (81)	284 (19)			
Clinic Group			*15.94*	*3*	*0.001*
Cardiology/Neurology	722 (83.9)	139 (16.1)			
Oncology	511 (76.6)	156 (23.4)			
Orthopaedics	283 (84)	54 (16)			
Obstetrics & Gynaecology	364 (79)	97 (21)			

*n = 67 missing; **n = 24 missing

### Demographic characteristics

A total of 1486 (76.3 %) of 1947 participants were asked to indicate their willingness and preferences for participating in future research through the HMRI Consumer Register. The remaining participants (*n* = 461) were called into their appointment before completing these questions (incomplete data not included in analysis). The demographic characteristics of participants are provided in Table 2. Participants were an average of 53 years of age and the majority were female (61.8 %). Most participants were married or living with their partner (65.5 %), had year 10 or lower level of education (47.2 %), did not have private health insurance (61.2 %) but did have a health care card (62.3 %). Of the total sample, 5.7 % reported being of Aboriginal and/or Torres Strait Islander origin.

**Table 2:** Demographic characteristics for individuals who completed the willingness to be contacted for future research question (*n* = 1486)

Demographic characteristics	Mean (SD)
Age	53.1 (19)
	N (%)
Gender	
Male	539 (37.0)
Female	900 (61.78)
Marital Status	
Single, never married	232 (15.6)
Married/Living with partner	959 (65.5)
Separated or divorced	167 (11.2)
Widowed	112 (7.5)
Education	
Year 10/School Certificate or lower	701 (47.2)
Higher School Certificate	210 (14.1)
Diploma/Trade Certificate	300 (20.2)
Bachelor Degree	164 (11.0)
Postgraduate Degree	87 (5.9)
Aboriginal and/or Torres Strait Islander Origin	
Yes	85 (5.7)
Possesses private health insurance	
Yes	577 (38.8)
Possesses healthcare card	
Yes	926 (62.3)
Clinic recruited from:	
Cardiology	256 (17.2)
Neurology	331 (22.3)
Oncology	348 (23.4)
Orthopaedics	207 (13.9)
Obstetrics & Gynaecology	336 (22.6)

### Consent and preferences for research participation

A total of 1032 of 1486 individuals (69.4 %) agreed to be contacted about future research opportunities. Table 3 outlines the preferred frequency of contact and acceptable modes of contact for the participants who agreed to be contacted about future research opportunities. Individuals were more likely to consent to be contacted about future research if they were older (*p* < 0.001). No other significant differences were found. Overall, 30.1 % were willing to be contacted once over a 12 month period, 29.5 % were willing to be contacted twice, and 38.7 % were willing to be contacted three or more times. The most acceptable mode of contact to issue an invitation to participate was email (50.3 %), followed by mailed letter (26.2 %). Telephone contact, either mobile or home line, was acceptable to 18.2 % and 16.8 % of the sample respectively. The majority of participants (88.5 %) selected only one preferred mode of contact.

**Table 3:** Preferred frequency and acceptable mode(s) of contact over 12 month period for register consenters (*n* = 1032)

Preferences for participation	N (%)
Preferred frequency of contact in next 12 months^a^	
One contact	311 (30.1)
Two contacts	304 (29.5)
Three contacts	164 (15.9)
Four contacts	91 (8.8)
Five contacts	15 (1.45)
Six or more contacts	129 (12.5)
Acceptable mode(s) of contact^b^	
Email	519 (50.3)
Mobile phone	188 (18.2)
Home phone	173 (16.8)
Posted letter	270 (26.2)
Number of contact options selected	
1	888 (88.5)
2	93 (9.3)
3	16 (1.6)
4	7 (0.7)

^a^1.7 % of participants did not complete these questions. Less than 2 % missing data

^b^Participants were able to select more than one mode of contact and therefore proportions exceed 100 %

### Feasibility

#### Cost per person recruited to the register

Based on opportunity cost, the set up and operation of the register in its first year required total resources valued at AU$47,719. When the useful life of fixed costs is considered, the annualised opportunity cost reduces to $37,187. In its first year of operation the register successfully recruited 1032 patients who were willing to participate in future research. This gives an opportunity cost per person recruited of $36 (based on annualised opportunity cost). The total monetary outlay in the registers first year of operation was $33,535. After amortising fixed costs, the annualised outlay is $23,004 or $22 per person recruited to the register. This indicates the use of volunteers contributes to an annual saving of $14,183.

#### Recruiter success measured by consent rates

A pool of 24 individuals, six research assistants and 18 volunteers, completed patient recruitment. The majority of data collectors were female (83 %), and the average age was 46 years (SD = 18.6). The time spent recruiting ranged from 2.5 to 94 h (M = 25.6; SD = 23.8). Consent rates per number approached varied from 54 % to 96 %. On average, research assistants performed more hours (M = 53.4, SD = 28.1) than volunteers (M = 16.4, SD = 11.0) and recruited a higher number of participants (research assistants: M = 156.8, SD = 96.6; volunteers: M = 41.2, SD = 24.2). To determine possible associations between recruiter characteristics and success rates, results from the simple linear regression are presented in Table 4. The type of recruiter (research assistant vs volunteer) showed some evidence of an effect on recruitment rates with research assistants having a higher recruitment rate compared to volunteers (mean difference = 8.67 %, 95 % CI: 0.51,16.82, *p* = 0.037), and explained 11 % of the variation in recruitment rates. This effect reduced to 6.8 % (95 % CI:-6.8,20.5, *p* = 0.332) in the multivariable model (results not shown).

**Table 4:** Results from univariate and multivariable linear regression models for data collector success as measured by consent rates. Multivariable models are adjusted for all variables in the table

			Univariate			Multivariable	
Variable	Class	Mean (95 % CI)	Absolute difference (95 % CI)	*P*	R-squared	Adjusted difference (95 % CI)	*P*
Gender	Female	76.4 (70.5, 82.3)	ref				
	Male	73.0 (71.3,74.8)	−3.4 (−9.5,2.7)	0.273	1.3 %	0.14 (−8.3.,8.6)	0.974
Medical setting experience	No	74.1 (68.9,79.3)	ref				
	Yes	78.3 (69.5,87.1)	4.2 (−4.0,12.5)	0.317	3.4 %	3.9 (−8.5,16.3)	0.535
Type	Volunteer	73.7 (68.9,78.5)	ref				
	RA	82.3 (76.2,88.5)	8.7 (0.5,16.8)	0.037	11.1 %	6.8 (−4.7,18.3)	0.247
Age	Per year		−0.1 (−0.5,0.2)	0.385	5.1 %	−0.1 (−0.3,0.2)	0.630

#### Completion rates

Of the 1947 participants who agreed to complete the touchscreen survey, 461 individuals (23.7 %) did not complete the willingness to participate in future research question. Of the 1032 participants who were willing to participate in future research, a further 18 individuals (1.7 %) did not complete one of three questions regarding their preferred frequency and mode of contact. Of the 2417 individuals approached, the proportion of individuals who were successfully enrolled onto the register (i.e. willing to participate and provided complete data on preferences) was 42 % of the sample.

## Discussion

This study examined the feasibility of developing a Consumer Register for accessing a sample of patients in a timely and cost effective manner according to their preferences. The HMRI Consumer Register aims to reduce costs, increase access to recently diagnosed patients, be inclusive of multiple chronic health conditions, encourage community participation and facilitate medical research in an era of consumer-driven health care. Overall, the study highlights that the HMRI Consumer Register is a promising alternative to population-based and traditional hospital - based registries.

### Consent rates

A large sample of outpatients across a range of chronic disease services were approached to indicate willingness and preferences for participating in a research register. Overall, 80.6 % of patients were willing to complete a touchscreen survey while attending appointments at a hospital-based outpatient clinic, which is comparable with consent rates for tablet-based surveys conducted in general practices and other health care settings [30, 31]. This demonstrates electronic surveys as an acceptable method of register recruitment in hospital outpatient clinics.

### Willingness to participate

Overall, 69.4 % of those who answered the question about willingness to be part of the register were willing to be part of a register and be contacted to be invited to participate in future research. The majority were willing to be contacted two or more times with opportunities for involvement in research. Email was the preferred method of contact which would provide researchers with a feasible and cost-effective way to communicate with potential participants. Previous research has indicated that, while population-based registries are a promising method of participant research recruitment, a lack of patient knowledge about the register and uncertainty of how their contact details were gained by researchers may lead to lower levels of consent [2, 32]. Additional barriers that prevent researchers from directly contacting patients, such as physician notification processes and screening, have also been suggested as causes of lower than the desirable consent rates frequently encountered [20]. These gaps were addressed in the current study given the direct patient recruitment at health services. This approach showed a reasonably high opt-on rate to a consumer-focussed register, however it was slightly lower than that of similar registries [23, 24].

### Cost

The high cost of register recruitment can often deter researchers from utilising this method. While the cost of recruiting from a population - based register can often be over $100 per patient, the annual opportunity cost of the HMRI Consumer Register is $36 per recruited patient. After amortising fixed costs, the annual financial outlay is $23,004 or $22 per person recruited to the register. This cost is similar to that found in a similar study of a Canadian ‘permission to contact’ database involving more than 7000 participants [24]. While demonstrating the financial benefit from volunteer labour, this cost-effective rate would reduce further with increased use of volunteers for data collection, rather than the mix of volunteers and paid employees used in this study. This cost outcome is a compelling indicator of an affordable approach to participant recruitment. However, these cost savings must be balanced against the characteristics of the participants on each register; it may be the case that recruitment from a population register may be more cost-effective when individuals with specific characteristics (for example, individuals at a specific time since diagnosis) are desired.

### Recruitment

Volunteer data collectors provided more than 300 h of recruitment assistance. However, it is important to note that employed research assistants had a higher consent rate than volunteers. Research assistants were employed using a more selective process (i.e. competitive application and interview rounds) and had a higher incentive to perform well due to their paid position, which may have influenced this difference. Research assistants also completed more hours and recruited a higher number of participants to the study which may have resulted in overall higher consent rates due to a greater opportunity to develop recruitment skills. A more rigorous selection criteria and acceptance process for volunteers may have helped boost consent rates. These factors should be considered by researchers when considering paid versus volunteer data collectors in future research projects.

### Enrolment

A considerable proportion of the sample (24.6 %) were unable to complete the survey prior to being called into their appointment and as such enrolment onto the HMRI Consumer Register was not finalized. This includes the 461 participants who did not receive the willingness to participate in future research item and an additional 18 participants who did not indicate a preferred frequency of contact. While this is a limitation of the recruitment approach within outpatient clinics, the benefits of recruiting patients at point of care included access to a sample of health service users with a wide range of chronic diseases, and instant data availability via electronic data collection. The completion rates may also be lower for this study as it was conducted in conjunction with another project in which a proportion of patients completed additional survey modules assessing preferences for health initiatives. These benefits and limitations should be carefully considered by future researchers planning on actively recruiting from healthcare services.

#### Implications for future research

While this paper reports data from patient recruitment within a hospital setting, there is potential to extend this recruitment approach into primary care and community health settings. Such an initiative is underway in Scotland through the Scottish Health Research Register [33]. This will provide scope for recruiting patients who may be seeking care for less acute or severe health conditions, and will provide access to a wide cross section of the community. It may also be possible to monitor their progress over time, providing potentially valuable information not only about their health care utilisation patterns but also their perceptions about these experiences. Previous research suggests that consent rates in primary care and community health care settings should be equal to or higher than those obtained in the current study [34].

A significant benefit of our approach is the use of touchscreen tablet computers for data collection. Most survey research benefits from an initial iterative process where a small sample of the data of interest is collected and analysed to ensure the aims of the study can be adequately answered. The data collected can then be modified if needed before undertaking further data collection. This iterative process is often difficult to do if recruiting from an established register. Registries are heavily dependent on collecting information from healthcare providers and medical records, making it difficult to make changes to the information requested without significant negotiations and subsequent training. Changing the data collected from patients is often a prolonged process, and has the potential to lead to errors in the data collected. Utilising a touchscreen tablet data collection approach means, however, that the information requested from participants can be quickly and easily changed at minimal cost. It is possible to modify the data collected from patients or add small modules to the existing core questions. This increases the specificity of data available to researchers requesting access to patients for research.

Gaining information about patient samples from current registries requires researchers to follow official channels by submitting time consuming requests to database owners. The HMRI Consumer Register is designed to facilitate researcher useability by employing an online database that researchers can independently query to ascertain sampling information. Researchers can obtain summary data about: the number of available participants who have indicated a willingness to be involved in research, their demographic data, health service and clinic attended and recent conditions. Future iterations will allow further refinement and advanced searches of this database with the goal to facilitating health research.

#### Limitations

These findings must be considered in light of several limitations. Firstly, there were high non-completion rates as a result of participants being called into their appointment before finishing the survey. This raises some questions about the utility of this approach to patient recruitment in clinics with quick patient throughput. Secondly, as patients completed the survey in the presence of a volunteer or research assistant, social desirability may have influenced patient responses. However, the touchscreen computer method of administration was utilised to mediate the effects of this as they are shown to increase privacy for participants [31]. Finally, it is important to acknowledge that intent does not always match action and while the majority of participants indicated willingness for their details to be entered onto a research register, this does not necessarily indicate that high response rates will result from studies which recruit participants from the register. The proportion of register participants who agree to participate in future research studies remains to be tested.

## Conclusions

Patient-centred registries are a feasible and acceptable approach to participant recruitment in research. A Consumer Register holds many benefits for both patients and researchers while maintaining close alignment with standards of patient centred health care values. This study is a preliminary proof of concept and further investigation of follow up participation rates and uptake will be assessed. Future research should be focussed on examining the consent rates of participants willing to be contacted with research opportunities and further developing the online database for researchers to utilise register participants.
